# The Use of Virtual Therapy in Cardiac Rehabilitation of Male Patients with Coronary Heart Disease: A Randomized Pilot Study

**DOI:** 10.3390/healthcare10040745

**Published:** 2022-04-16

**Authors:** Sandra Jóźwik, Adam Wrzeciono, Błażej Cieślik, Paweł Kiper, Joanna Szczepańska-Gieracha, Robert Gajda

**Affiliations:** 1 Faculty of Physiotherapy, University School of Physical Education in Wroclaw, 51-612 Wroclaw, Poland; j_sandra@wp.pl (S.J.); awp97adam@wp.pl (A.W.); joanna.szczepanska@awf.wroc.pl (J.S.-G.); 2 Department of Kinesiology and Health Prevention, Jan Dlugosz University in Częstochowa, 42-200 Częstochowa, Poland; gajda@gajdamed.pl; 3 Physical Medicine and Rehabilitation Unit, Azienda ULSS 3 Serenissima, 30126 Venice, Italy; pawel.kiper@aulss3.veneto.it; 4 Center for Sports Cardiology at the Gajda-Med Medical Center in Pultusk, 06-102 Pultusk, Poland

**Keywords:** virtual reality, cardiac rehabilitation, mental health

## Abstract

The study aimed to evaluate the effectiveness of virtual reality therapy (VRT) in the treatment of anxiety–depressive disorders and in reducing stress levels in a group of men with coronary heart disease (CHD) participating in cardiac rehabilitation (CR). The study included 34 men with CHD who were assigned to the experimental group (EG) or the control group (CG). CR in the EG was supported by 8 VRT sessions, while CR in the CG was supplemented with 8 SAT sessions. Anxiety–depressive disorders were assessed using the Hospital Anxiety and Depression Scale (HADS). Perceived stress was assessed using the Perception of Stress Questionnaire (PSQ). In the EG, all measured parameters improved after the intervention. Significant reductions in HADS total score, the HADS-A, general stress score, emotional tension, and the external stress were obtained. In the CG, a deterioration in all measured parameters was observed. Significant changes were obtained in the general stress score and intrapsychic stress. The analysis between groups showed that the effectiveness of psychological interventions significantly differed between groups. The study results confirmed that supplementing standard CR with VRT leads to an improvement in the mental state of the patients and thus has a positive effect on the course of CR. However, the small sample size and high withdrawal rate prompt cautious interpretation of the results.

## 1. Introduction

Depression, anxiety, and high levels of perceived stress are increasingly common problems associated with cardiovascular disease (CVD) and occur in one in five patients with coronary heart disease (CHD), peripheral arterial disease (PAD), and heart failure (HF) [1]. The psychopathologies that occur lead to an increase in mortality and increased costs of health services, as well as to a decrease in quality of life and the chances of returning to work [2,3,4]. Depression is two to three times more common in patients with CHD than in the general population, and the incidence of generalized anxiety disorder (GAD) in patients with CHD ranges from 11% to 14% [5]. Depression is considered an independent factor that increases the risk of incidents and deaths and is a strong predictor of subsequent hospitalizations, whereas anxiety appears to be such a predictor only in conjunction with depressive symptoms [3,4].

The relationship between anxiety–depressive disorders and the risk of CVD is confirmed by behavioral and physiological mechanisms. Behavioral factors are primarily related to the inability to adhere to health-related guidelines that could prevent CVD (i.e., regular physical activity, quitting smoking, and the use of cardiovascular medications as recommended) and emphasize the relationship between CVD, depression, and a poor health prognosis. Anxiety–depressive disorders can affect the following physiological reactions: inflammatory processes, autonomic nervous system dysfunctions, and abnormal coronary blood flow. All of these factors directly increase the risk of myocardial ischemia [5,6].

Available evidence suggests that comorbidity related to depressive disorders and anxiety disorders is substantial and widespread [7]. In most cases, anxiety precedes the onset of depression disorders and appears to be a risk factor for the development of major depression [8]. Anxiety–depressive disorders coexisting with CVD affect both men and women [3]. Research shows that men and women react differently to psychological symptoms due to a difference in behavior, sympathetic nervous system activity, and activation of the hypothalamic–pituitary–adrenal axis (HPA). In women with depression, frequent somatic symptoms are observed, whereas inflammation and an increased number of white blood cells are more common in men [9,10,11]. Jankowska et al. highlighted other factors that influence depression among older men. Due to the low level of androgens that modify the functioning of the central nervous system (also responsible for the regulation of mood), men are prone to depressive symptoms and, consequently, to a reduction in the quality of life [12].

The most important risks in people with CVD and anxiety–depressive disorders, as comorbidities, are an increased risk of recurrent cardiovascular events and increased mortality. Therefore, reducing perceived stress and anxiety–depressive disorders should be one of the main goals of cardiac rehabilitation (CR). Meanwhile, these disorders are still not sufficiently diagnosed and, consequently, are not adequately treated. As a result, this contributes to the lack of the desired effects of rehabilitation. As standard care, traditional cardiac rehabilitation is supplemented with relaxation techniques or Schultz autogenic training (SAT). In the age of technological development, modern technologies, including virtual reality (VR), are increasingly supporting the treatment of anxiety and depression symptoms. Literature reviews clearly show the effectiveness of VR therapy in the treatment of psychiatric disorders [13,14]. The beneficial effect of VR therapy in reducing the level of anxiety and depression has been shown in chronic obstructive pulmonary disease and CHD patients [15,16]. Additionally, in cardiology, studies indicate that patients are happy to use various forms of training and report a desire to continue exercise programs after the completion of CR [17].

This study aimed to assess the impact of VRT on reducing perceived stress levels and depression and anxiety symptoms in a group of men with coronary heart disease participating in CR.

## 2. Materials and Methods

### 2.1. Participants

**Figure 1:**
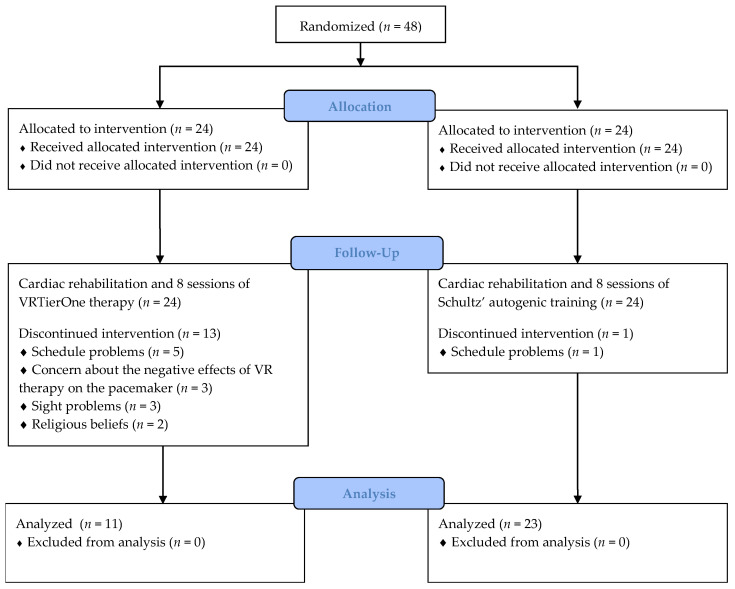
The flow diagram.

The study enrolled 48 men diagnosed with CHD who met the inclusion criteria. The characteristics of the study group are presented in Table 1. Participants were recruited between January and April 2020, from the PRO CORDE Cardiology Center in Wroclaw (Poland) and were randomly divided (ratio 1:1) into two groups: 24 patients were assigned to the experimental group (EG) and 24 patients to the control group (CG). Randomization was carried out using Research Randomizer, a web-based service that offers random assignment. The inclusion criteria were as follows: male gender, age 40–85 years, diagnosed CHD, and undergoing second phase of CR in ambulatory conditions. Exclusion criteria included: female gender; a cognitive impairment that prevented the self-completion of research questionnaires (Mini-Mental State Examination score of <24), disturbances of consciousness, psychotic symptoms, bipolar disorder, and other serious psychiatric disorders; the initiation of psychiatric or psychological treatment during the study; patient refusal at any stage of the study; and contraindications to the application of VRT (i.e., epilepsy, dizziness, and visual disturbances). All participants voluntarily participated in the study giving their written informed consent. The patients were also aware of the exact course of the study and informed that they could withdraw from participation at any stage without consequences. Thirteen participants from the EG did not complete the study due to vision problems, the fear that VR may adversely affect the operation of their pacemaker, religious beliefs, or organizational problems (the VR therapy schedule conflicted with the patients’ home responsibilities). One person dropped-out from the CG due to scheduling issues. Finally, 34 men participated in the study, with 11 in the EG and 23 in the CG (Figure 1). The study participation rate was 70.8%. The study protocol was approved by the Institutional Review Board of the University School of Physical Education in Wroclaw (Poland) (Resolution No. 31/2019). The study was carried out in accordance with the Declaration of Helsinki 1975, revised Hong Kong 1989.

### 2.2. Interventions

Experimental group: Patients in the EG (*n* = 11), in addition to standard CR, participated in 8 therapeutic sessions, two times a week for four weeks with the use of the VRTierOne device (Stolgraf, Stanowice, Poland). The therapy was based on the metaphor of a virtual therapy garden, where the patient was supposed to calm down and relax. The therapy aimed to silence the overactive sympathetic part of the autonomic nervous system, reduce the level of stress, improve mood, evoke positive associations, and activate the patient’s participation in cardiac rehabilitation. During therapy, the patients were sitting in a chair and wearing a helmet with VR goggles that enabled the image to be displayed in high resolution and with a high refresh rate (90 Hz), and they had manipulators in their hands that allowed them to perform tasks in the virtual therapeutic garden. The visual effects were complemented by surround sound. The same VR system was described in earlier articles [16,18,19].

Control group: Patients in the CG (*n* = 23), in addition to standard CR, participated in 8 sessions of Schultz autogenic training (SAT), the standard method of psychological support for CVD patients, performed two times a week for four weeks. Therapy was conducted by a psychologist. Relaxation was based on six basic exercises. At first, the feeling of heaviness (i.e., heavy arms, heavy legs) followed by the feeling of warmth (i.e., warm hands, warm legs) was evoked. Subsequently, the work of the heart (calm heart) and breathing (relaxed breathing) were regulated. At the end, the feeling of warmth in the solar plexus followed by the feeling of a cool forehead were evoked. Relaxation was played from a CD player by a psychologist. The aim of the therapy was general relaxation and reducing anxiety and sadness, as well as supporting the treatment of depression and sleep problems.

Standard cardiac rehabilitation: Additionally, all patients participated in a four-week outpatient CR. At first, each patient had an echocardiographic exercise test as a criterion to start CR. Based on the exercise test, the individual maximum heart rate (HRmax) during exercise was selected for each patient. Patients exercised for 80 min 3 times a week at an intensity level of 60% to 85% of HRmax. The CR started with a 40-minute cycle ergometer training session. Subsequently, the patients participated in 40 min of group general fitness exercises or 40 min cardio fitness training, which consisted of exercises performed on a treadmill, rowing machine, multi-gym, or elliptical trainer. During the CR, the heart rate of the patients was continuously monitored and their blood pressure was measured before and after the training.

### 2.3. Outcome Measures

Two standardized questionnaires were used in the study: The Hospital Anxiety and Depression Scale (HADS) and the Perception of Stress Questionnaire (PSQ). In addition, a self-administered questionnaire on sociodemographic, clinical, and lifestyle data was used. The questionnaire was developed by the authors. The questions concerned the following information: education, marital status, employment, type of disease, the coexistence of diabetes, as well as lifestyle, subjective assessment of health, and the ability to cope with stress. The data collected were compared with the information contained in the medical records. Baseline assessment was performed in all patients before CR and after four weeks of rehabilitation.

Hospital Anxiety and Depression Scale: The Polish version of the HADS was used to assess the level of anxiety and depression in patients. The questionnaire consists of 14 questions rated from 0 to 3, with 3 representing the highest level of anxiety or depression. In the HADS, two separate subscales give partial scores for anxiety (HADS-A) and depression (HADS-D). The score ranges from 0 to 42, with higher scores indicating the most severe symptoms of anxiety and depression [20]. A score of 8 out of 21 on each subscale is considered the cut-off point, which means that patients who score 8 or more on at least one subscale are classified into the group with varying degrees of anxiety or depressive disorder. The normal values are 0–7, while 8–10 indicates mild anxiety or depressive disorders. Values in the range from 11 to 21 are assumed to indicate severe anxiety or depressive disorders. A literature review based on 747 articles showed that obtaining a minimum of 8 points gives the highest specificity and sensitivity of the test. The Cronbach alpha reliability coefficient for HADS-A was α = 0.83 and for HADS-D was α = 0.82 [21].

Perception of Stress Questionnaire: The Perception of Stress Questionnaire (PSQ) by Plopa and Makarowski consists of 27 statements that allow the level of emotional tension, external stress, intrapsychic stress, and the risk of lying to be determined. During examination, patients answered the questions using a five-point Likert scale (true, rather true, hard to say, rather not true, not true). Interpreting the overall score ranging from 21 to 105 points allows the patient’s stress level to be determined, where higher results indicate more severe symptoms of stress. The cut-off point is a score of 60 points. The authors of the questionnaire obtained Cronbach’s alpha reliability coefficients at the level: α = 0.72 for external stress, α = 0.81 for emotional tension, and α = 0.69 for intrapsychic stress [22].

### 2.4. Data Analysis

All statistical analyses were performed using Statistica 13 software (StatSoft, Cracow, Poland). Baseline characteristics were reported as mean with standard deviation (SD) for continuous variables and percentages for categorical variables. Chi-square tests were used to assess significant associations between categorical variables and the Mann–Whitney U test was used for continuous variables. As normality tests (Shapiro-Wilk test) revealed that none of the outcome measures followed a normal distribution, nonparametric tests were used. Differences between variables within groups were compared using the Wilcoxon signed-rank test. Differences between the two groups were assessed with the Mann–Whitney U test. The effect sizes (ES) were determined by Morris effect size d [23] and classified as follows: 0.1–0.3, small effect; 0.3–0.5, intermediate effect; and ≥0.5, strong effect [24]. The level of statistical significance was established at α = 0.05.

## 3. Results

Thirty-four participants were included in the analysis of results. There were no significant differences between the EG and the CG in terms of the parameters that describe the study group (Table 1). In the baseline assessment of measured outcomes, the mental state of participants in the experimental and control groups did not statistically significantly differ (*p* > 0.05) (Table 2).

**Figure 2:**
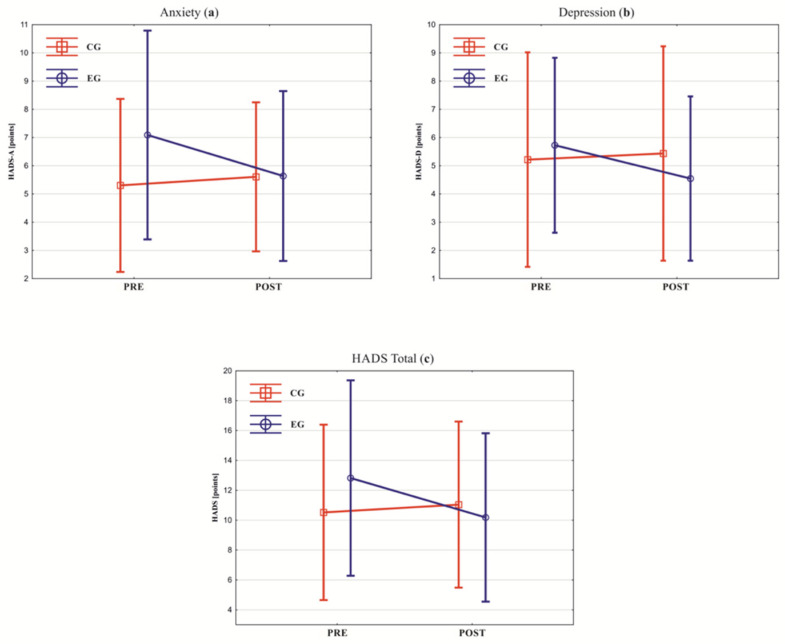
Between-group analysis of the HADS (Hospital Anxiety and Depression Scale) questionnaire scores in pre- and post-rehabilitation: (**a**) anxiety, (**b**) depression, and (**c**) total score. CG—control group; EG—experimental group. Squares and circles show the mean, vertical lines show standard deviations, and slanted lines show the direction of between-group measurements change of the mean values.

**Figure 3:**
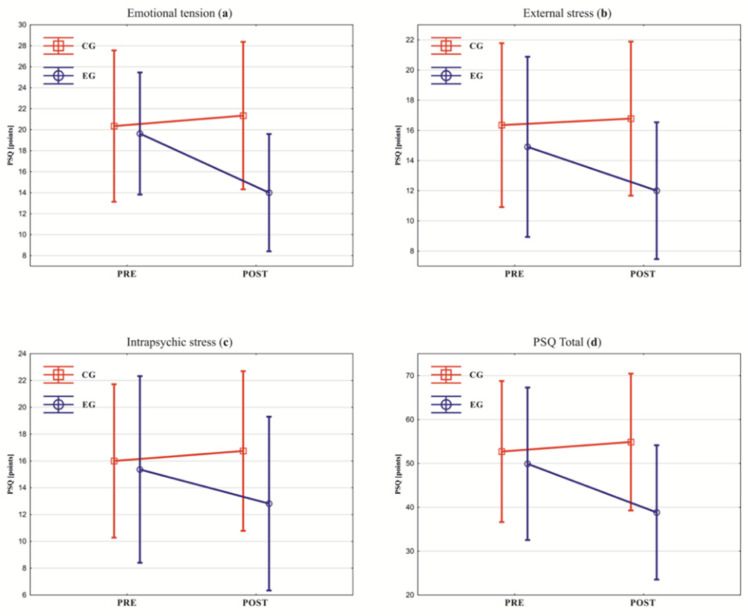
Between-groups analysis of the PSQ questionnaire scores in pre- and post-rehabilitation: (**a**) emotional tension; (**b**) external stress; (**c**) intrapsychic stress; and (**d**) total score. CG—control group; EG—experimental group; PSQ—Perception of Stress Questionnaire. Squares and circles show the mean, vertical lines show standard deviations, and slanted lines show the direction of between-group measurements change of the mean values.

In the EG, an improvement in all tested parameters was observed (Figure 2 and Figure 3). Significant reductions in the measured parameters were obtained in the HADS total score (*p* = 0.04), HADS-A (*p* = 0.02), general stress score (*p* = 0.01), emotional tension (*p* = 0.002), and external stress (*p* = 0.03). For all the significant changes, the estimated ES was strong (Table 3). In the CG, deterioration was observed in the mean values of all studied parameters (Figure 2 and Figure 3). Significant deterioration was observed in overall stress score (*p* = 0.005) and intrapsychic stress (*p* = 0.02). For all the significant changes, the estimated ES was strong (Table 3). The between-groups analysis showed that the effectiveness of psychological interventions significantly differed between the EG and the CG (*p* < 0.05) (Figure 2 and Figure 3). The estimated ES was negative and strong (for general stress score, emotional tension, external stress, and intrapsychic stress) or negative and intermediate (for HADS total score, HADS-A, and HADS-D) (Table 3).

## 4. Discussion

This study evaluated the effect of the use of VRT on changes in the severity of anxiety–depressive symptoms and the level of stress in a group of men participating in CR. The statistical analysis of the results confirmed the hypothesis that relies on the reduction in perceived stress and the alleviation of depression and anxiety symptoms. The values of the measured parameters showed a significant reduction in the HADS total score, HADS-A, general stress score, emotional tension, and external stress with strong ES. Furthermore, the following measured parameters significantly deteriorated in the CG: general stress score and intrapsychic stress with strong ES. In addition, a comparison of the effectiveness of VRT versus SAT indicated a significantly greater effectiveness of VRT, with intermediate or strong ES. The results can suggest VRT’s potential to become an important therapy in supplementing CR, while the SAT has ceased to fulfil its purpose.

Previous studies have shown the importance of managing mental health in cardiac patients. Goldstein et al. pointed out that people with depression-anxiety disorders are more prone to cardiovascular events and have an increased risk of mortality [25]. Attention is drawn to the need to perform psychosomatic disorders screening and create new and effective treatment methods as a complement to existing methods [26,27]. Szczepańska-Gieracha et al. indicated that standard CR is not an effective method of treating depression-anxiety disorders in cardiology patients. Furthermore, the early diagnosis and treatment of psychosomatic disorders in cardiological patients is necessary [28]. Thus, possible solutions can be found in rapidly developing modern technology [29].

VRT is widely used in psychology, especially as a therapeutic intervention for anxiety, depression, and stress reduction. This determined the use of VRT in our study. Cieślik et al. noted that most studies concern the use of VR in the reduction in anxiety symptoms, and all studies (23 reviews with 12,991 participants overall) described the positive impact of using VR in the treatment of anxiety disorders [13]. Yeung et al. described the beneficial effect of using immersive VR to cope with a spectrum of emotional problems, such as depression and anxiety disorders [30]. In earlier work, we confirmed the effective use of VRT in cardiac patients in reducing the symptoms of anxiety and depression and the positive effect on the course of CR [16].

Our results are in line with those of Garcia-Bravo et al. The study concerned the use of VR and video games in patients with ischemic heart disease in the second phase of CR. The results showed that supplementing CR with VR therapy can slightly reduce the severity of depression symptoms, thus improving quality of life, exercise tolerance, and resistance to fatigue [31]. On the other hand, Vieira et al. did not show any changes in the severity of depression symptoms after the VR intervention in patients participating in CR [32]. The lack of effectiveness of the CR intervention could have been caused by the fact that the authors based this only on the feedback exercise without psychotherapeutic elements.

The available literature indicates that the problem of anxiety–depressive disorders is more frequently affecting women [33,34]. In our opinion, this may result from the failure to consider psychological needs based on gender differences. Another important problem is the reluctance of men to seek help in the mental sphere. In our previous study, we conducted a similar intervention in women with heart disease. The result indicated that VRT is an effective support method in reducing stress symptoms [35]. In the current study, we are concerned about the large group of patients who dropped out. The most common reason was scheduling problems. The percentage of those who refused to participate in VRT was significantly higher in the male group compared to the female group in our previous study [35]. This issue should be investigated more, because it seems that it is more common for men to decline treatment of mental health problems.

Staiger et al. attribute such dependence with imposed stereotypes about masculinity. Depression is a problem that causes shame in men because it is often accompanied by a feeling of helplessness, a lack of control, and weakness [36]. Therefore, the presented factors may cause disproportions in studies’ results, resulting from the fact that women seek help more often. Jbilou et al. described the emotional problems that often occur in patients with mitral regurgitation. The authors argue that the most at-risk group for psychological and social disorders are men who have problems with speaking and expressing their emotions and avoid seeking help [37]. Another major problem for men with CVD is erectile dysfunction, which can also affect their mental health [38]. The factors presented can predispose men to suppressing their emotions and thus to the development of cardiovascular diseases, which led us to investigate male patients.

Stress has been identified as a major risk factor for CVD and stroke [39]. Acceleration of the occurrence of a cardiovascular or cerebrovascular event is explained by changes related to stress in the balance of the sympathetic and parasympathetic systems, as well as in the activation of the hypothalamus–pituitary–adrenal axis [40]. As a result, heart rate and blood pressure increase and heart rate variability is reduced. Long-term physiological stress responses may lead to clinical effects (e.g., myocardial infarction, acute coronary syndrome, and atrial fibrillation), resulting in persistent pathophysiological effects such as myocardial ischemia or cardiac electrical instability [41]. The most justified reason seems to limit stressors and their negative impact on health. However, due to their multitude and numerous connections with the fast-paced lifestyle that is present nowadays, the implementation of such solutions seems practically impossible. Therefore, appropriate psychological care for cardiac patients should be applied to limit subsequent cardiovascular events. Comprehensive CR for CVD patients often includes psychological treatment. The review by Richards et al. suggests that psychological intervention could reduce cardiac mortality. However, the available trials are of low quality and no unequivocal conclusions can be drawn [42].

### 4.1. Practical Implications

The results obtained in the presented study and our previous studies [16,19,35] encourage reflection and the consideration of introducing changes in the treatment of cardiac patients with anxiety–depressive disorders. In patients who were subjected to standard CR, the examined mental state parameters increased. The results suggest that CR combined with SAT is an insufficient method for treating anxiety–depressive disorders in cardiac patients [43]. First, the presence of a qualified psychotherapist in an interdisciplinary therapeutic team is very often overlooked. Therefore, therapeutic sessions are only played from CDs, and patients are only made aware of the seriousness of mental disorders in cardiac disorders. Second, SAT was developed in the 1960s. It is supposed that this method may not be effective due to the lifestyle of modern people who are exposed to a large number of stimuli every day. As a result, there is a need for a therapy that involves patients as much as possible. Due to the immersion phenomenon, VR offers such an approach. However, due to the high withdrawal rate due to patients’ concerns about the impact of VR on pacemakers or vision problems, we should look for alternative support methods for this kind of patient.

### 4.2. Limitations

The study also has several limitations. First, a small group of patients participated in the study. Furthermore, some of the EG participants did not complete the study due to concerns about the effects of VR on pacemaker operation, which prompts reflection on the possible use of other modern technologies to manage the mental health of cardiac patients. Secondly, no follow-up measurements were performed, which precludes assessment of the long-term effects of VR application. The mentioned limitations encourage a cautious interpretation of the presented research and indicate possible directions in which research in this area should be continued.

## 5. Conclusions

The presented results confirm that VRT is an attractive modality that has great potential to support CR by reducing the level of perceived stress and anxiety–depressive symptoms. Additionally, VRT was shown to be an effective alternative to standard SAT. Thus, combined cardiological rehabilitation with virtual therapy could have the potential to reduce the risk of further cardiovascular events. However, the study group was small, so conclusions should be treated with caution. The high percentage of patients who resigned from participation in VR therapy should encourage the identification of other methods of psychological support that do not cause as many concerns and problems in cardiac patients.

## Figures and Tables

**Table 1:** Participants baseline characteristics.

Variable	Total	Experimental Group	Control Group	*p*
*n*	34	11	23	-
Age, years (*SD*)	63.82 (8.13)	66.55 (9.63)	62.52 (7.18)	0.18
Body mass, kg (*SD*)	86.15 (11.99)	82.36(11.47)	87.96 (12.05)	0.21
Height, cm (*SD*)	174.50 (6.30)	172.73 (6.57)	175.35 (6.12)	0.36
BMI, kg/cm2 (*SD*)	28.26 (3.33)	27.61 (3.44)	28.57 (3.31)	0.21
**Specific diagnosis, *n* (%)**				
PCI	9 (26)	4 (36)	5 (22)	0.53
STEMI	7 (21)	3 (27)	4 (17)	
NSTEMI	11 (32)	2 (18)	9 (39)	
CABG	4 (12)	1 (9)	3 (13)	
Heart stimulator	1 (3)	0 (0)	1 (9)	
Ablation	1 (3)	0 (0)	1 (9)	
Paroxysmal atrial fibrillation	1 (3)	1 (3)	0 (0)	
**Education, *n* (%)**				
Primary/vocational	13 (38)	4 (36)	9 (39)	0.80
Secondary	8 (24)	2 (18)	6 (26)	
Higher	13 (38)	5 (45)	8 (35)	
**Marital status, *n* (%)**				
Married	28 (82)	9 (82)	19 (83)	0.83
Single	4 (12)	1 (9)	3 (13)	
Widowed	2 (6)	1 (9)	1 (4)	
**Employment status, *n* (%)**				
Employed	17 (50)	5 (45)	12(52)	0.45
Disability pension	1 (3)	1 (9)	0 (0.00)	
Retired	15 (44)	5 (45)	10 (43)	
Unemployed	1 (3)	0 (0.00)	1 (4)	
**Subjective assessment of health status, *n* (%)**				
Good	2 (6)	0 (0)	2 (9)	
Average	20 (59)	8 (73)	12 (52)	0.41
Bad	12 (35)	3 (27)	9 (39)	
**Managing stress, *n* (%)**				
Yes	19 (56)	7 (64)	12 (52)	
No	5 (15)	1 (9)	4 (17)	0.76
Hard to say	10 (29)	3 (27)	7 (31)	
**Voluntary physical activity, *n* (%)**				
Regular	13 (38)	6 (55)	7 (30)	
Occasional	15 (44)	5 (45)	10 (43)	0.13
None	6 (18)	0 (0)	6 (27)	
**Diet adherence, *n* (%)**				
Yes	12 (35)	3 (27)	9 (39)	
Mostly	18 (53)	7 (64)	11 (48)	0.69
No	4 (12)	1 (9)	3 (13)	

BMI—body mass index; PCI—percutaneous coronary intervention; STEMI—ST-segment elevation myocardial infarction; NSTEMI—non-ST segment elevation myocardial infarction; CABG—coronary artery bypass grafting.

**Table 2:** Baseline measurement of outcomes.

	GROUP		
	Experimental (*n* = 11)	Control (*n* = 23)	
Variables	Median [IQR]	Median [IQR]	*p*
HADS	15.00 [9.00–17.00]	10.00 [7.00–16.00]	0.26
HADS-A	8.00 [3.00–10.00]	6.00 [4.00–8.00]	0.12
HADS-D	6.00 [3.00–8.00]	4.00 [2.00–9.00]	0.72
General stress score	46.00 [39.00–62.00]	50.00 [39.00–65.00]	0.64
Emotional tension	19.00 [17.00–24.00]	22.00 [13.00–27.00]	0.83
External stress	16.00 [9.00–18.00]	15.00 [11.00–20.00]	0.42
Intrapsychic stress	14.00 [10.00–23.00]	15.00 [11.00–22.00]	0.80

IQR—interquartile range; HADS—Hospital Anxiety (A) and Depression (D) Scale.

**Table 3:** The analysis of outcomes.

	Within-Group Analysis								Between-Group Analysis	
	Experimental Group				Control Group					
Variables	Pre Median [IQR]	Post Median [IQR]	*p* *	Effect Size	Pre Median [IQR]	Post Median [IQR]	p*	Effect Size	Δ Post–pre *p* †	Effect Size
HADS	15.00 [9.00–17.00]	11.00 [3.00–15.00]	**0.04**	0.73	10.00 [7.00–16.00]	10.00 [8.00–16.00]	0.10	−0.36	**0.02**	−0.41
HADS-A	8.00 [3.00–10.00]	6.00 [2.00–8.00]	**0.02**	0.83	6.00 [4.00–8.00]	6.00 [3.00–8.00]	0.23	−0.29	**0.005**	−0.48
HADS-D	6.00 [3.00–8.00]	6.00 [1.00–7.00]	0.07	0.55	4.00 [2.00–9.00]	4.00 [2.00–9.00]	0.18	−0.26	**0.02**	−0.40
General stress score	46.00 [39.00–62.00]	36.00 [27.00–48.00]	**0.01**	0.96	50.00 [39.00–65.00]	54.00 [41.00–70.00]	**0.005**	-0.65	**<0.001**	−0.66
Emotional tension	19.00 [17.00–24.00]	14.00 [9.00–17.00]	**0.002**	1.21	22.00 [13.00–27.00]	21.00 [15.00–28.00]	0.06	−0.43	**<0.001**	−0.67
External stress	16.00 [9.00–18.00]	12.00 [8.00–14.00]	**0.03**	0.76	15.00 [11.00–20.00]	17.00 [13.00–21.00]	0.11	−0.34	**<0.001**	−0.55
Intrapsychic stress	14.00 [10.00–23.00]	11.00 [7.00–16.00]	0.09	0.50	15.00 [11.00–22.00]	16.00 [11.00–22.00]	**0.02**	−0.52	**0.01**	−0.43

Bold highlights statistical significance *p* < 0.05; IQR—interquartile range; HADS—Hospital Anxiety (A) and Depression (D) Scale; *—*p*- value for within-group analysis (Wilcoxon signed-rank test); †—*p*-value for between-group analysis (Mann–Whitney U test); Δ post–pre—the difference between final and baseline measurement compared between groups.

## Data Availability

Anonymous data will be available upon request and stored in the repository of the University School of Physical Education in Wroclaw.
